# Patterns of health-promoting behaviors and associated factors in family caregivers of people receiving cancer treatment: A latent class profile analysis

**DOI:** 10.1002/pon.6145

**Published:** 2023-05-08

**Authors:** Elisa H. Son, Gwenyth R. Wallen, Sharon Flynn, Li Yang, Lena J. Lee

**Affiliations:** 1 Translational Biobehavioral and Health Disparities Branch, National Institutes of Health (NIH) Clinical Center, Bethesda, Maryland, USA; 2 National Heart, Lung, and Blood Institute, NIH, Bethesda, Maryland, USA

**Keywords:** caregivers, health behavior, health promotion, neoplasms, psycho-oncology

## Abstract

**Objective:**

Family caregivers tend to neglect their health while prioritizing the needs of their care recipients. Identifying subgroups of caregivers based on the patterns of health-promoting behaviors (HPBs) may help develop tailored interventions for them, yet little is known. The purpose of this study was: (1) to identify latent classes with distinct patterns of HPBs in family caregivers of people with cancer; and (2) to investigate factors associated with the latent class membership.

**Methods:**

We performed a cross-sectional data analysis using the baseline dataset from a longitudinal survey study that assessed HPBs of family caregivers of individuals who received cancer treatment at a national research hospital (*N* = 124). Latent class profile analysis was conducted to identify latent classes based on the subdomains of the Health-Promoting Lifestyle Profile II, followed by multinomial logistic regression analysis to investigate factors associated with the latent class membership.

**Results:**

Three latent classes were identified: a high level of HPB (Class 1, 25.8%); a moderate level of HPB (Class 2, 53.2%); and a low level of HPB (Class 3, 21.0%) of HPBs. Controlling for caregiver age and sex, caregiver burden due to lack of family support, perceived stress, self-efficacy and body mass index were factors associated with the latent class membership.

**Conclusions:**

HPBs of our caregiver sample appeared in relatively stable patterns at different levels. Higher caregiver burden and perceived stress and lower self-efficacy were associated with the lower practice of HPBs overall. Our findings may serve as a reference for screening caregivers who need support and developing person-centered interventions.

## 1 | INTRODUCTION

Approximately 48 million individuals served as unpaid caregivers in the United States (U.S.) in 2020. Caring for someone receiving cancer treatment is one of the main reasons to become a caregiver.^1^ Compared to non-cancer caregiving, caring for a family member with cancer requires more hours of caregiving with greater complexity of tasks that include daily living support, care coordination, and nursing tasks (e.g., catheter care, tube feedings).^2^ Caregiving can be a rewarding experience, but family caregivers of people with cancer often suffer from chronic stress and multiple stress-related symptoms that include depression, anxiety, fatigue, sleep disturbance, and cognitive impairment.^3,4^

The burden of caregiving may disrupt family caregivers’ health-promoting behaviors (HPBs) as they tend to prioritize the needs of the care recipient while neglecting their own needs.^5,6^ Understanding HPBs in cancer caregivers is important because caregiver participation in health-promoting activities may directly impact the health and well-being of the individuals with cancer as well as the caregivers.^6^ According to Pender et al.’s (2011)^7^ Health Promotion model, the likelihood of engaging in HPBs is influenced by individual characteristics (e.g., age, gender, education, stress) and behavior-specific cognitions (e.g., self-efficacy). The outcome of Pender’s model is the likelihood of engaging in HPBs, including health responsibility (e.g., asymptomatic screening, routine healthcare), physical activity, nutrition, spiritual growth (e.g., feeling of harmony, finding a sense of purpose), interpersonal relations, and stress management.^7,8^ Caregiving responsibilities can make it difficult for caregivers to practice HPBs.^5,9^ Indeed, compared to non-caregiver controls, caregivers are less physically active, eat an imbalanced diet, participate less in leisure activities and social relations, experience difficulty in developing inner resources, and have difficulty attending medical visits for their health when necessary.^10–12^ Previous studies have reported that hours of caregiving, psychological factors such as depression and anxiety, and the level of self-efficacy were associated with caregivers’ practice of HPBs.^10,13,14^ Limited practice of HPBs can lead to lifestyle- and stress-related diseases, such as cardiometabolic disease.^15,16^ However, research on family caregivers’ HPBs in the cancer context has been relatively limited.

A person-centered approach focusing on the similarities or relationships among individuals with the purpose of identifying individuals who share common characteristics (e.g., cluster analysis, latent class analysis [LCA]) may be preferred in health behavior research. Many studies, however, used a variable-centered approach that focuses on examining the relationships between variables of interest (e.g., regression, factor analysis).^10–12,17–19^ A variable-centered approach limits the translation of findings to individuals since it fails to capture the diverse nature of the sample and could lead to over-generalized conclusions regarding the population.^17,18^ The goal of a person-centered approach is to divide a heterogenous sample into homogeneous subgroups, in which members are similar to each other while different from individuals in other subgroups within a given population based on a set of chosen variables.^17–19^ LCA provides a superior approach to discover homogeneous unobserved subgroups (i.e., latent classes) that exist within a heterogenous sample using multiple observed indicators.^20^ LCA may be useful for identifying meaningful subgroups of caregivers based on their similarities on a set of HPBs. The identified latent classes can be used as an important outcome for us to explore the characteristics of caregivers with different health behavior patterns.

To date, there have only been a few studies that investigated patterns of health behaviors in populations other than family caregivers (e.g., people at high risk of stroke, midlife women, older adults).^21–23^ These studies often included dichotomized indicators of physical activity, diet, smoking, and alcohol consumption. The patterns of HPBs in family caregivers may differ from those found in other populations because they are in unique circumstances where they dedicate considerable time and energy to caregiving and thereby practice a lack of self-care overall.^5,6^ Understanding the patterns of HPBs using indicators that reflect comprehensive aspects of self-care in family caregivers can guide us to identify potential behavioral risks that may affect their health and develop strategies to reduce those risks. The purpose of this study was: (1) to identify latent classes with distinct patterns of HPBs in family caregivers of people with cancer; and (2) to investigate factors associated with the identified latent class membership.

## 2 | METHODS

### 2.1 | Study design and participants

This cross-sectional data analysis focused on evaluating HPBs of family caregivers of individuals who received cancer treatment at the National Institutes of Health (NIH) Clinical Center. The current analysis was part of a prospective repeated measures survey research titled, the Web-based Patient-Reported Outcome Measurement Information System (PROMIS^®^) to explore Burden and Stress in Cancer Caregivers (BaSiC^2^; NCT#01981538). Eligible adult (≥18 years) family caregivers (*N* = 129) of people who initiated new cancer treatment at the NIH Clinical Center participated in online surveys over three time points (baseline, 3 months, and 6 months) between March 2014 and July 2016. The baseline dataset of the original research was used for the current analysis. Of 129 caregiver participants who submitted the baseline data, five participants who did not enter information about their baseline HPBs were excluded. Therefore, selected data from the baseline dataset obtained from 124 participants were included in the current analysis. Further details regarding the study procedures and participant descriptions of the original research are published elsewhere.^24^

### 2.2 | Ethical considerations

The institutional review boards of the National Heart, Lung, and Blood Institute at the NIH approved the original research. Written informed consent was obtained from all caregiver participants before beginning any study procedures.

### 2.3 | Measures

#### 2.3.1 | Sample characteristics

Demographic characteristics of family caregivers included age, sex, race/ethnicity, marital status, employment status, annual household income, body mass index (BMI), and presence of chronic health problems. The participants’ caregiving characteristics included relationship with the care recipient, caregiver role, days of caregiving per week, and hours of caregiving per day. Care recipient characteristics were age, sex, primary disease, and type of cancer treatment.

#### 2.3.2 | Health-promoting behaviors

HPBs were measured using the Health-Promoting Lifestyle Profile II (HPLP-II).^25^ The HPLP-II measures the frequency of self-reported participation in HPBs represented by six subdomains: health responsibility (9 items), physical activity (8 items), nutrition (9 items), spiritual growth (9 items), interpersonal relationships (9 items), and stress management (8 items). A total of 52 individual items are scored using a 4-point Likert scale from 1 (never) to 4 (routinely), with higher scores indicating more frequent participation in HPBs. We score each subdomain by calculating the mean of the assigned items. The HPLP-II is a well-validated, reliable measure that has been widely used in diverse adult populations, including caregivers.^10,12,21^ Cronbach’s alpha of the subdomains in this analysis ranged from 0.75 to 0.88.

#### 2.3.3 | Caregiver burden

Caregiver burden was measured using the Caregiver Reaction Assessment (CRA).^26^ The CRA is a 24-item scale to assess the impact of caregiving on the following five subdomains: caregiver esteem (7 items), impact on finances (3 items), impact on health (4 items), impact on schedule (5 items), and lack of family support (5 items). Each item is rated on a 5-point Likert-type response from 1 (strongly disagree) to 5 (strongly agree). The subdomains are scored by calculating the mean of the items for each subdomain, with a range of 1–5. Higher subdomain scores indicate greater burden, with the exception of caregiver esteem; a higher score means higher caregiver esteem. The CRA is a valid and reliable measure for use in cancer caregivers.^27^ Cronbach’s alpha of the subdomains in this analysis ranged from 0.68 to 0.86.

#### 2.3.4 | PROMIS and NIH Toolbox measures

Perceived stress, self-efficacy, and psychological factors were measured using the PROMIS and NIH Toolbox.^28–30^ The PROMIS and NIH Toolbox are highly reliable and validated measures of self-reported health outcomes: the PROMIS assessing physical, mental, and social well-being and the NIH Toolbox assessing cognitive, emotional, sensory, and motor functions. In the current analysis, we used the PROMIS measures to assess symptoms of depression and anxiety, and the NIH Toolbox measures to assess perceived stress, self-efficacy, and loneliness. Except for self-efficacy, items of each concept were rated on a 5-point Likert scale ranging from 1 to 5, with higher scores indicating higher levels of the concept being measured. Items of self-efficacy were rated on a 4-point Likert scale ranging from 1 to 4, with higher scores indicating higher levels of self-efficacy. The measures of depression and anxiety were delivered using computer adaptive testing (CAT), which typically administers 4–7 items per concept. Raw scores derived from CAT are standardized to *T*-scores that are normed to the general population with a mean of 50 and standard deviation (*SD*) of 10. Perceived stress, self-efficacy, and loneliness were measured using a 10-item, 10-item, and 5-item fixed-form questionnaire, respectively. For these concepts measured using the PROMIS and NIH Toolbox, scores that are 1 *SD* or more below the mean indicate low levels of the concept being measured, and scores that are 1 *SD* or more above the mean indicate high levels of the concept being measured.

### 2.4 | Statistical methods

For each variable, descriptive analysis was performed including mean, *SD*, frequency, and percentage. LCA, a type of finite mixture model, was used to divide our sample into homogenous subgroups (i.e., latent classes) using multiple indicators. It is termed LCA when the indicators are categorical variables, and it is termed latent class profile analysis (LCPA) when the indicators are continuous variables.^20^ We performed LCPA via the maximum-likelihood with robust standard errors estimation method to identify latent classes of family caregivers with distinct patterns of HPBs based on six subdomains of HPLP-II: health responsibility, physical activity, nutrition, spiritual growth, interpersonal relationships, and stress management. We selected the subdomains of HPLP-II as indicators of LCPA because the measure assesses comprehensive aspects of HPBs based on Pender’s Health Promotion model^7,8^ and has been used consistently in caregiver research.^10,12^ LCPA was estimated by iteratively adding potential classes to determine how many classes best describe the patterns observed in our data and the best model that fits the sample. The Akaike information criterion (AIC), Bayesian information criterion (BIC), and sample-size adjusted BIC (SABIC) are commonly used model selection criteria that reflect how well the model predicts the data, with smaller values being better. Entropy, a measure of class separation, indicates how well the classes are differentiated; closer to 1 is preferred and above 0.8 is acceptable. The Vuong–Lo–Mendell–Rubin likelihood ratio test (VLMR-LRT) and Lo-Mendell-Rubin adjusted LRT (LMR-LRT) evaluate whether the log-likelihood value of *k* class model is better than that of *k* − 1 class model (*p* < 0.05). The selection of the final model was made by a comprehensive review of the aforementioned fit indices as well as interpretability and clinical meaning of the latent classes. Multivariate analysis of variance (MANOVA) and post hoc tests were performed to analyze the differences in HPBs between the latent classes.

Following LCPA, we performed multinomial logistic regression analysis to identify factors that are associated with the latent classes. Bivariate analyses were performed to examine the impact of the following factors on the latent class membership: caregiver characteristics (marital status, employment status, annual income, BMI, presence of chronic health problems, relationship with care recipient, caregiver role, hours of caregiving per day), care recipient characteristics (age, sex), NIH Toolbox perceived stress, five subdomains of caregiver burden (CRA caregiver esteem, CRA impact on finances, CRA impact on health, CRA impact on schedule, and CRA lack of family support), NIH Toolbox self-efficacy, NIH Toolbox loneliness, PROMIS anxiety, and PROMIS depression. Factors with *p* < 0.10 in the bivariate analyses were included in the multiple multinomial logistic regression model. Collinearity between the factors was assessed. Factors in the final model were selected using backward elimination with a removal criterion of *p* ≥ 0.10. A priori known factors, caregiver’s age and sex were controlled in the model. Descriptive analysis and multinomial logistic regression analysis were conducted using IBM SPSS Version 28,^31^ and LCPA was conducted using Mplus Version 8.6.^32^

## 3 | RESULTS

### 3.1 | Demographics and descriptive characteristics

Demographic and descriptive characteristics are presented in Table 1. The mean age of our caregiver sample (*N* = 124) was 48.98 years (*SD* = 11.69), ranging from 20 to 76 years. The majority of them were female (68.5%), white (80.6%), non-Hispanic (86.2%), married or living with a partner (83.7%), and working either full-time or part-time (73.4%). Slightly less than half of them were spouse caregivers (49.2%) and had an annual household income of more than $89,000 (47.0%). The mean BMI was 26.95 kg/m^2^ (*SD* = 5.48), and more than half of them had at least one chronic health problem (52.0%). Less than half of them were sole primary caregivers (46.0%), providing the mean of 5.83 days (*SD* = 2.11) per week and the mean of 10.13 h (*SD* = 7.99) per day of caregiving. The mean scores of NIH Toolbox perceived stress, NIH Toolbox loneliness, PROMIS depression, and PROMIS anxiety were within 1 *SD* of the score normed for the general population, and the mean score of NIH Toolbox self-efficacy was comparable to the normed-level. Of the subdomains of caregiver burden, CRA impact on schedule and CRA impact on finances were the two highest levels of burden.

The mean age of care recipients was 42.03 years (*SD* = 18.25; range, 6–76), and less than half of them were female (46.8%). Carcinoma (50.0%) and leukemia (21.8%) were the two most reported primary diagnoses, and biotherapy/immunotherapy (64.5%) was the most reported treatment type.

### 3.2 | Latent classes for HPBs

Based on the fit indices of LCPA (Table 2), 1-to 4-class models were compared, and the following results were confirmed: (1) the AIC, BIC, and SABIC of the 3-class model were smaller than those of the 2-class model and were similar to those of the 4-class model, meaning that the 3-class and 4-class models predict the data better than the 2-class model; (2) the entropy of the 3-class model was greater than that of the 2-class and 4-class models, meaning that the classes in the 3-class model are better differentiated compared to the 2-class and 4-class models; and (3) the VLMR-LRT and LMR-LRT of the 3-class model were significant, whereas those of the 2-class and 4-class models were not, meaning that the 3-class model is statistically better than the 2-class and 4-class models. The 3-class model was selected by comprehensively reviewing the fit indices, clinical meaningfulness, and interpretability.

**FIGURE 1:**
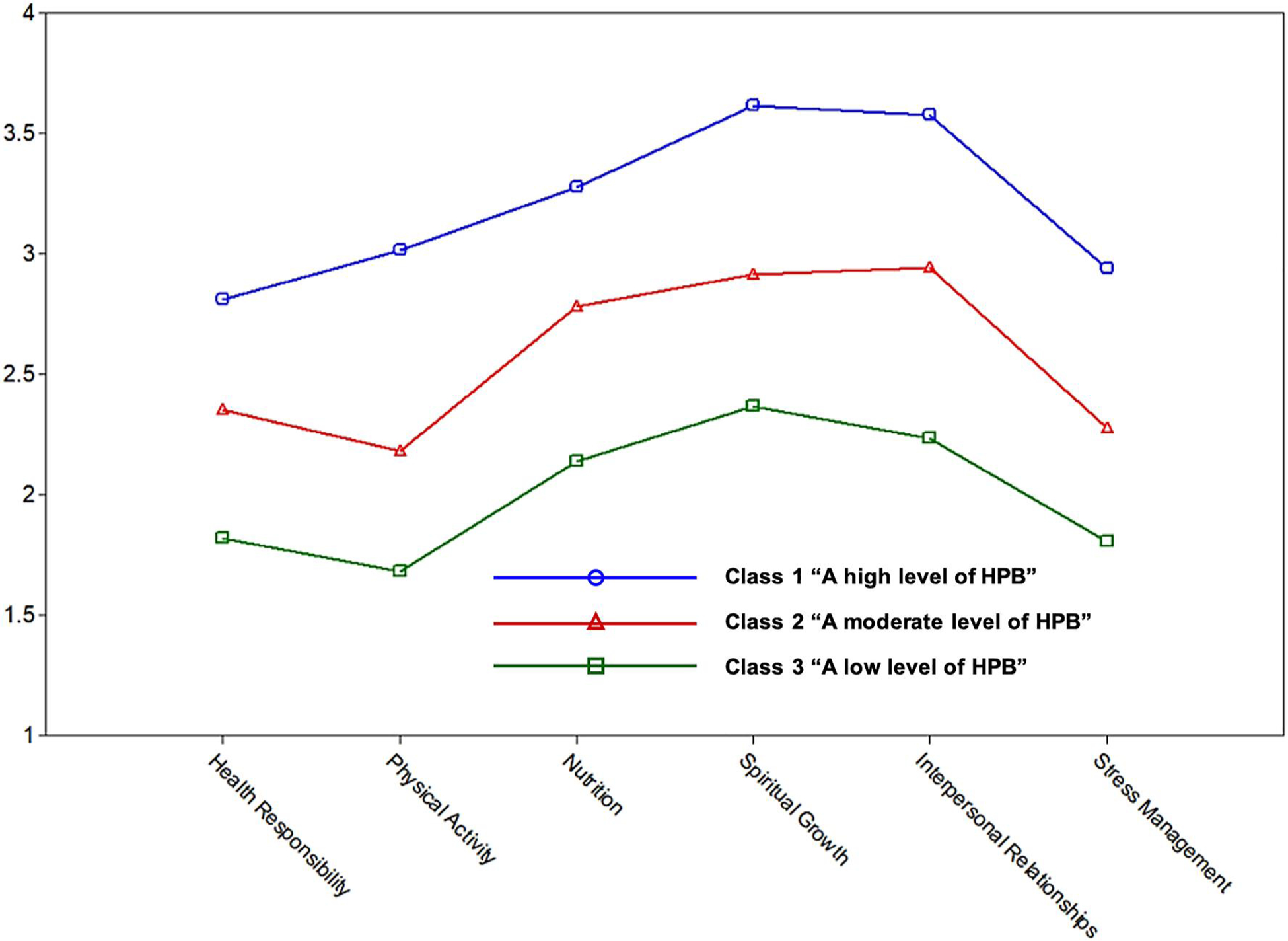
Latent classes based on the subdomains of HPLP-II. HPB, health-promoting behavior; HPLP-II, health-promoting lifestyle profile II.

The three latent classes were labeled based on the level of each subdomain of HPLP-II (Figure 1). Class 1, labeled “a high level of HPB” (*n* = 32, 25.8%), showed an overall high level of HPBs, except that taking health responsibility and stress management were slightly lower than the other subdomains. Class 2, labeled “a moderate level of HPB” (*n* = 66, 53.2%), showed a lower level of HPBs overall than Class 1. Class 3, labeled “a low level of HPB” (*n* = 26, 21.0%), showed the lowest level of HPBs overall among the three latent classes. In both Class 2 and Class 3, physical activity was the lowest subdomain, followed by stress management and health responsibility.

MANOVA showed significant differences in the subdomains of HPLP-II between the three latent classes: *F* (12, 230) = 31.145, *p* < 0.001; Wilks’ Lambda = 0.145. Each univariate ANOVA revealed that all six subdomains of the HPLP-II differed significantly between the three latent classes. Post hoc tests were performed using Dunnett T3 for the health responsibility subdomain (due to violation of the equal variance assumption) and Bonferroni for the other subdomains. Post hoc tests showed that: Class 2 and Class 3 had lower mean scores for all subdomains than Class 1; and Class 3 also had lower mean scores for all subdomains than Class 2 (see Table 3).

### 3.3 | Associated factors of latent classes for HPBs

Thirteen factors (annual income, BMI, presence of chronic health problems, NIH Toolbox perceived stress, NIH Toolbox self-efficacy, NIH Toolbox loneliness, PROMIS depression, PROMIS anxiety, CRA lack of family support, CRA impact on finances, CRA impact on schedule, CRA impact on health, care recipient age) with *p* < 0.10 in the bivariate analyses and control variables (caregiver age and sex) were included in the multiple multinomial logistic regression model for the latent class membership, setting Class 1 “a high level of HPB” as the reference. Based on the removal criterion of *p* ≥ 0.10, nine factors (annual income, presence of chronic health problems, NIH Toolbox loneliness, PROMIS depression, PROMIS anxiety, CRA impact on finances, CRA impact on schedule, CRA impact on health, care recipient age) were removed from the final model. No significant multi-collinearity between the factors existed. The final results of the multiple multinomial logistic regression analysis are presented in Table 4. CRA lack of family support, NIH Toolbox perceived stress, and caregiver BMI were factors significantly associated with the membership of Class 2 “a moderate level of HPB” versus Class 1 “a high level of HPB.” Caregivers who reported higher burden due to lack of family support were more likely to be in Class 2 (odds ratio [OR] = 2.834, 95% confidence interval [CI] = 1.136–7.067) versus Class 1. Caregivers with higher perceived stress were more likely to be in Class 2 (OR = 1.108, CI = 1.020–1.203) versus Class 1. Caregivers with higher BMI were more likely to be in Class 2 (OR = 1.163, CI = 1.034–1.309) versus Class 1.

CRA lack of family support, NIH Toolbox perceived stress, NIH Toolbox self-efficacy, caregiver sex, and BMI were factors significantly associated with the membership of Class 3 “a low level of HPB” versus Class 1 “a high level of HPB.” Caregivers who reported higher burden due to lack of family support were more likely to be in Class 3 (OR = 3.763, CI = 1.277–11.088) versus Class 1. Caregivers with higher perceived stress were more likely to be in Class 3 (OR = 1.183, CI = 1.063–1.318) versus Class 1. Caregiver with higher self-efficacy were less likely to be in Class 3 (OR = 0.880, CI = 0.789–0.981) versus Class 1. Compared to male caregivers, female caregivers were less likely to be in Class 3 (OR = 0.113, CI = 0.021–0.596) versus Class 1. Caregivers with higher BMI were more likely to be in Class 3 (OR = 1.258, CI = 1.087–1.455) versus Class 1.

## 4 | DISCUSSION

This study identified latent classes with distinct patterns of HPBs in family caregivers of people with cancer and investigated factors associated with the identified latent class membership. Three latent classes were identified in our caregiver sample: Class 1, “a high level of HPB”; Class 2, “a moderate level of HPB”; and Class 3, “a low level of HPB.” While the overall level of HPBs significantly differed between the three latent classes, the levels of the three subdomains (health responsibility, physical activity, and stress management) were relatively lower than those of the other subdomains (nutrition, interpersonal relationships, spiritual growth) in all latent classes. As family caregivers prioritize their care recipients ahead of their own needs, they generally lack the time and energy to take care of their health.^5,6^ Our finding supports previous findings suggesting that caregiving responsibilities prevent caregivers from attending medical appointments or participating in leisure activities to relieve stress.^5,9^ This also concurs with previous studies indicating that cancer caregivers commonly had the lowest engagement in physical activity, and they were relatively active in interpersonal relationships and spiritual growth.^10,12^ Caregivers seem to prefer behaviors that give them emotional strength and motivation during cancer treatment without requiring much time.^10,12^ Further research is needed to develop and test simple interventions that do not require excessive time and resources from the caregivers. Our analysis showed relatively stable patterns of HPBs at different levels, indicating that the subdomains of HPBs might be related to each other. The approach of focusing on patterns of HPBs may provide useful insights leading to the development of tailored and effective interventions that can target multiple HPBs simultaneously.

This study demonstrated that LCPA is beneficial in identifying a specific group of caregivers who practice low levels of HPBs. Class 3, which showed the lowest level of HPBs among the three latent classes, accounted for 21.0% of our sample. Studies have reported that caregivers are vulnerable to adverse health outcomes influenced by elevated stress hormones and altered immune and metabolic function.^15,33^ Caregivers are particularly at risk of developing cardiometabolic disease such as type 2 diabetes and coronary artery disease.^15,16^ Behavioral factors, including lack of physical activity, unbalanced diets and poorly managed stress, are known to contribute to this risk in general.^34^ Due to the burden, stress, and lack of time related to caregiving responsibilities, caregivers are more likely to have such behavioral risk factors compared to non-caregiver controls.^11,12^ In our analysis, caregivers in Class 3 may be at increased risk for unfavorable health outcomes, including the incidence of cardiometabolic disease, as they had the lowest levels of HPBs. Special attention is needed for this group of caregivers.

The results of this study provide further evidence regarding factors associated with the patterns of HPBs in cancer caregivers. In our analysis, caregivers with higher perceived stress, a higher burden due to lack of family support, and lower self-efficacy were more likely to engage in lower levels of HPBs. Male caregivers and caregivers with higher BMI in our sample were also associated with practicing lower levels of HPBs. Although evidence found in cancer caregivers is still lacking, our findings align with the literature explaining that: males, higher levels of burden, stress perceived by caregivers, and lower self-efficacy were associated with decreased HPBs.^9,35,36^ The finding about gender difference is consistent with research on caregivers of childhood cancer survivors,^37^ indicating male sex was associated with a higher risk of engaging in unhealthy behaviors, including physical inactivity, cigarette smoking, and alcohol consumption. The findings may be explained by socialized gender roles and gender differences in stress-coping strategies.^37^ This study also confirmed the findings of other caregiving studies,^9,38,39^ that self-efficacy was associated with participation in HPBs. In line with Akpinar et al.,^40^ our results indicated that caregivers who lack family support might feel more burdened with care responsibilities, leading to poorer self-care. Investigating associated factors may guide us in determining caregivers in need of supportive interventions.

### 4.1 | Clinical implications

Oncology clinical care staff may serve as health coaches to encourage family caregivers to take care of their health. As oncology clinical care staff interact with caregivers during cancer treatment, they are in a key position to assess the burden and distress experienced by caregivers and screen those in need of additional support. We recommend paying the closest attention to caregivers with high levels of perceived stress, high levels of burden due to lack of family support, and low levels of self-efficacy. Through counseling, clinical staff can more accurately assess and understand the caregivers’ current status and support their needs. They may recommend simple stress relief activities, respite care or social work services, and community caregiving resources to caregivers. In particular, a growing number of researchers are focusing on technology-assisted interventions that family caregivers can use without time and space restrictions.^41^ Technology-assisted interventions for caregivers are becoming more diverse, including web-based education, mobile app-based mindfulness meditation, online support groups, and relaxation in virtual environments.^41,42^ Participating in such interventions might have positive effects on alleviating the burden and stress experienced by caregivers, which might lead to their active engagement in HPBs. Consistent with the literature,^14^ self-efficacy was another significant factor explaining engagement in HPBs. Supporting caregivers to set and achieve attainable health goals might improve their self-efficacy^43^ and ultimately increase the chances of engaging in more sustainable healthy behaviors.

### 4.2 | Study limitations

This study has several limitations. This study only included family caregivers of people receiving cancer treatment at the NIH Clinical Center, a unique clinical research hospital setting that provides care to those enrolled in clinical trials with no charge to the research participants. The findings of this study may not be generalizable to caregivers of people receiving care in general hospitals. Another limitation was that the nature of the cross-sectional design did not allow us to determine whether the association between each risk factor and the latent class membership was causal. For example, perceived stress may worsen HPBs, which then exacerbates levels of perceived stress. Future research is needed to determine whether the process is unidirectional or cyclical. In addition, the sample size (*N* = 124) in the current analysis was relatively small considering that the number of parameters in LCPA was 26. Further studies using a large, population-based sample are recommended to confirm the findings of the present study.

## 5 | CONCLUSION

Using LCPA, this study identified three latent classes with distinct patterns of HPBs in family caregivers of people with cancer. Further, this study identified factors that may explain the likelihood of being in a particular class of HPBs. Our findings add to the evidence that researchers may refer to when identifying at-risk groups and developing person-centered intervention programs to improve HPBs in cancer caregivers. The current findings can also be used as a reference for oncology clinical care staff when screening caregivers who may need support in self-care to promote health. Future studies may consider integrating health-risk behaviors or predicting disease outcomes based on the patterns of HPBs.

## Figures and Tables

**TABLE 1:** Demographic and descriptive characteristics (*N* = 124).

Variables	*N* (%) *M* (SD), range
Caregiver characteristics	
Age in years	48.98 (11.69), 20.00–76.00
Sex	
Male	39 (31.5)
Female	85 (68.5)
Race	
White	100 (80.6)
African American	5 (4.0)
Asian	8 (6.5)
Other	11 (8.9)
Ethnicity	
Hispanic	17 (13.8)
Non-Hispanic	106 (86.2)
Marital status	
Married/living with a partner	103 (83.7)
Not married^a^	20 (16.3)
Employment status	
Full-time	71 (57.3)
Part-time	20 (16.1)
Not working^b^	33 (26.6)
Annual household income	
<$50,000	33 (28.7)
$50,000-$89,000	28 (24.3)
>$89,000	54 (47.0)
BMI, kg/m^2^	26.95 (5.48), 17.20–43.40
Chronic health problem	
Yes	64 (52.0)
No	59 (48.0)
Relationship with care recipient	
Spouse	61 (49.2)
Non-spouse family member^c^	60 (48.4)
Friend	3 (2.4)
Caregiver role	
Sole primary caregiver	57 (46.0)
Part of caregiving team	67 (54.0)
Caregiving days per week	5.83 (2.11), 1.00–7.00
Caregiving hours per day	10.13 (7.99), 1.00–24.00
Caregiver burden^d^	
Caregiver esteem	4.37 (0.51), 2.57–5.00
Lack of family support	1.86 (0.75), 1.00–4.40
Impact on finances	2.81 (1.15), 1.00–5.00
Impact on schedule	3.44 (0.86), 1.00–5.00
Impact on health	2.18 (0.75), 1.00–5.00
Perceived stress^e^	52.05 (9.85), 31.45–85.38
Self-efficacy^e^	51.68 (9.33), 17.33–68.43
Loneliness^e^	54.19 (10.63), 37.10–85.20
Depression^f^	52.44 (7.72), 34.17–81.83
Anxiety^f^	57.44 (6.78), 42.32–84.90
Care recipient characteristics	
Age in years	42.03 (18.25), 6.00–76.00
Sex	
Male	66 (53.2)
Female	58 (46.8)
Primary disease	
Carcinoma	62 (50.0)
Leukemia	27 (21.8)
Sarcoma	24 (19.4)
Lymphoma	9 (7.3)
Myeloma	1 (0.8)
Thymoma	1 (0.8)
Treatment type	
Biotherapy/immunotherapy	80 (64.5)
Allogeneic HSCT	12 (9.7)
Surgery	11 (8.9)
Chemotherapy	9 (7.3)
Combination therapy	8 (6.5)
Radiation therapy	4 (3.2)

*Note*: Numbers may not sum to total because of missing data.

Abbreviations: BMI, body mass index; HSCT, hematopoietic stem cell transplant.

a Not married = never married, separated or divorced, widowed.

b Not working = unemployed, retired, disabled, student and other.

c Non-spouse family member = parent, child, sibling, sibling-in-law, aunt.

d Caregiver burden was measured using the Caregiver Reaction Assessment.

e Perceived stress, self-efficacy, and loneliness were measured using NIH Toolbox.

f Depression and anxiety were measured using PROMIS.

**TABLE 2:** Fit indices of LCPA models.

Class	AIC	BIC	SABIC	Entropy	VLMR-LRT, *p*-value	LMR-LRT, *p*-value
1	1323.866	1357.709	1319.765	−	-	-
2	1122.597	1176.183	1116.104	0.808	0.1492	0.1553
3	1030.449	1103.777	1021.563	0.875	0.0070	0.0078
4	1017.596	1110.665	1006.318	0.860	0.1667	0.1751

Abbreviations: AIC, Akaike information criteria; BIC, Bayesian information criteria; LCPA, latent class profile analysis; LMR-LRT, Lo-Mendell-Rubin adjusted likelihood ratio test; SABIC, sample-size adjusted BIC; VLMR-LRT, Vuong-Lo-Mendell-Rubin likelihood ratio test.

**TABLE 3:** Differences in the subdomains of HPLP-II between latent classes.

	Total sample (*N* = 124)	Class 1 (n = 32, 25.8%) “a high level of HPB”	Class 2 (n = 66, 53.2%) “a moderate level of HPB”	Class 3 (*n* = 26, 21.0%) “a low level of HPB”		
Variables	*M* (*SD*)	*M* (*SD*)	*M* (*SD*)	*M* (*SD*)	*F*	Post hoc tests^a^
HPLP-II subdomains						
Health responsibility	2.36 (0.54)	2.81 (0.46)	2.36 (0.45)	1.82 (0.27)	38.772***	Class 2, Class 3 < Class 1; Class 3 < Class 2
Physical activity	2.29 (0.77)	3.03 (0.62)	2.19 (0.59)	1.65 (0.60)	40.440***	Class 2, Class 3 < Class 1; Class 3 < Class 2
Nutrition	2.78 (0.54)	3.28 (0.32)	2.78 (0.38)	2.13 (0.45)	66.407***	Class 2, Class 3 < Class 1; Class 3 < Class 2
Spiritual growth	2.98 (0.56)	3.61 (0.27)	2.92 (0.35)	2.37 (0.48)	88.045***	Class 2, Class 3 < Class 1; Class 3 < Class 2
Interpersonal relationships	2.96 (0.57)	3.58 (0.28)	2.94 (0.36)	2.23 (0.33)	120.156***	Class 2, Class 3 < Class 1; Class 3 < Class 2
Stress management	2.35 (0.55)	2.95 (0.46)	2.28 (0.34)	1.80 (0.36)	68.878***	2,3 < 1; 3 < 2

*Note*: All health-promoting behaviors are scored on a scale of 1–4. A higher score indicates practicing more health responsibility, more physical activity, more nutrition, more spiritual growth, more interpersonal relationships, and more stress management.

Abbreviations: HPB, health-promoting behavior; HPLP-II, health-promoting lifestyle profile II.

a In post hoc tests, Dunnett T3 was used for health responsibility, and Bonferroni was used for the other subdomains.

*** *p* < 0.001.

**TABLE 4:** Multiple multinomial logistic regression for latent classes.

Models	Variables	*B*	SE	Wald (χ^2^)	p-value	OR (95% CI)
Class 2 “a moderate	Intercept	−6.583	4.294	2.351	0.125	
level of HPB”	Caregiver burden—lack of family support	1.042	0.466	4.991	0.025	2.834 (1.136, 7.067)
	Perceived stress	0.103	0.042	5.956	0.015	1.108 (1.020, 1.203)
	Self-efficacy	−0.038	0.038	0.989	0.320	0.963 (0.895, 1.037)
	Caregiver age	−0.014	0.024	0.332	0.565	0.986 (0.942, 1.033)
	Caregiver sex (ref: male)	−0.897	0.640	1.963	0.161	0.408 (0.116, 1.430)
	Caregiver BMI	0.151	0.060	6.293	0.012	1.163 (1.034, 1.309)
Class 3 “a low	Intercept	−8.367	5.663	2.183	0.140	
level of HPB”	Caregiver burden—lack of family support	1.325	0.551	5.774	0.016	3.763 (1.277, 11.088)
	Perceived stress	0.168	0.055	9.405	0.002	1.183 (1.063, 1.318)
	Self-efficacy	−0.128	0.056	5.330	0.021	0.880 (0.789, 0.981)
	Caregiver age	−0.021	0.032	0.406	0.524	0.980 (0.920, 1.044)
	Caregiver sex (ref: male)	−2.179	0.848	6.612	0.010	0.113 (0.021, 0.596)
	Caregiver BMI	0.229	0.074	9.543	0.002	1.258 (1.087, 1.455)

*Note*: The reference category is: Class 1 “high level.” H0 versus H1: Likelihood ratio *χ*^2^ (df 12) = 67.814 (*p* < 0.001). Nagelkerke Pseudo-*R*^2^ = 0.488 (the model accounts for approximately 48.8% of the total variance).

Abbreviations: BMI, body mass index; HPB, health-promoting behavior.

## Data Availability

The data that support the findings of this study are available on request from the corresponding author, Dr. Lena J. Lee. The data are not publicly available due to their containing information that could compromise the privacy of research participants. Additionally, since the project was funded through the NIH Intramural Research Program, the NIH technically owns the data.
